# Design, Implementation and Evaluation of an Immersive Teleoperation Interface for Human-Centered Autonomous Driving

**DOI:** 10.3390/s25154679

**Published:** 2025-07-29

**Authors:** Irene Bouzón, Jimena Pascual, Cayetana Costales, Aser Crespo, Covadonga Cima, David Melendi

**Affiliations:** 1 CTIC Foundation, 33203 Gijón, Asturias, Spain; irene.bouzon@fundacionctic.org (I.B.); jimena.pascual@fundacionctic.org (J.P.); cayetana.costales@fundacionctic.org (C.C.); aser.crespo@fundacionctic.org (A.C.); covadonga.cima@fundacionctic.org (C.C.); 2 Department of Informatics, University of Oviedo, 33203 Gijón, Asturias, Spain

**Keywords:** teleoperation, autonomous vehicles, virtual reality, situational awareness, immersive interface, multimodal feedback, human-robot interaction, robot simulation, human-centered design

## Abstract

**Highlights:**

**What are the main findings?**

- This paper presents a novel VR-based teleoperation interface designed to support remote human intervention in autonomous-vehicle operations.
- Operators receive synchronized visual, auditory, and haptic feedback to facilitate timely and informed interventions in simulated emergency scenarios, bridging the sensory and control gaps between remote operators and the operational environment of the vehicle.
- The participants reported a perceived sense of safety, control, and satisfaction while using the immersive teleoperation system.

**What is the implication of the main findings?**

- Comprehensive multimodal feedback (such as visual, auditory, and haptic channels) is instrumental in supporting situational awareness without adding excessive cognitive load.
- The ability to interact directly in an immersive environment significantly contributes to enhancing trust in autonomous systems.

**Abstract:**

As autonomous driving technologies advance, the need for human-in-the-loop systems becomes increasingly critical to ensure safety, adaptability, and public confidence. This paper presents the design and evaluation of a context-aware immersive teleoperation interface that integrates real-time simulation, virtual reality, and multimodal feedback to support remote interventions in emergency scenarios. Built on a modular ROS2 architecture, the system allows seamless transition between simulated and physical platforms, enabling safe and reproducible testing. The experimental results show a high task success rate and user satisfaction, highlighting the importance of intuitive controls, gesture recognition accuracy, and low-latency feedback. Our findings contribute to the understanding of human-robot interaction (HRI) in immersive teleoperation contexts and provide insights into the role of multisensory feedback and control modalities in building trust and situational awareness for remote operators. Ultimately, this approach is intended to support the broader acceptability of autonomous driving technologies by enhancing human supervision, control, and confidence.

## 1. Introduction

As automated mobility technologies continue to evolve, the integration of autonomous systems into daily life presents both unprecedented opportunities and complex challenges. Connected Autonomous Vehicles (CAVs) promise to improve traffic efficiency, reduce accidents, and enhance accessibility. Despite their real-world deployment being limited by technical constraints and broader societal concerns, previous work has extensively described the deployment of unmanned ground vehicles (UGV) or unmanned aerial vehicles (UAV) in specific contexts, such as remote sensing, search and rescue, security and surveillance, manufacturing, precision agriculture, and civil infrastructure inspection [1]. Some of these autonomous systems often struggle to handle unpredictable or ambiguous scenarios, known as “edge cases”, that require contextual reasoning, moral judgment, or nuanced interpretation of human behavior. These limitations have sparked a growing consensus across academia, industry, and policy spheres regarding the need to incorporate human oversight into autonomous driving architectures.

In this context, human-in-the-loop systems have emerged as a key strategy for ensuring safety, accountability, and adaptability in autonomous driving [2]. These systems introduce a human decision-making layer capable of controlling in critical situations where autonomy alone may fall short or may be impossible. One of the most prominent applications of this approach is teleoperation, in which a remote human operator temporarily intervenes to guide the vehicle through complex or uncertain situations, such as navigating around ambiguous obstacles, responding to unpredictable mobility conditions, or resolving ethical dilemmas. This approach not only serves as a failsafe mechanism but also contributes to building public trust and ensuring that CAVs operate safely in dynamic environments.

Recent research has explored how immersive technologies, particularly virtual reality (VR), can improve teleoperation by enhancing the situational awareness and engagement of operators. VR allows remote users to perceive spatial information more naturally, visualize critical events in real time, and interact with control interfaces using intuitive gestures and movements. When combined with multimodal feedback (such as haptic, visual, or auditory cues), VR-based systems can support more responsive and informed decision-making. However, challenges persist, including managing latency, mitigating cognitive overload, and ensuring usability across different operator profiles.

This paper presents a novel VR-based teleoperation interface designed to support remote human intervention in autonomous-vehicle operations. The system integrates a high-fidelity simulation environment, a modular architecture for real-time data exchange, and a multimodal VR interface that supports both physical and gestural control. Operators receive synchronized visual, auditory, and haptic feedback to facilitate timely and informed interventions in simulated emergency scenarios, bridging the sensory and control gaps between remote operators and the operational environment of the vehicle. A preliminary user study was conducted to evaluate the system’s usability, effectiveness, and its impact on operator trust and perceived safety. Both subjective assessments (via questionnaires) and objective metrics (e.g., task success rate, collision frequency, and reaction time) were collected to inform future refinements of the system and contribute design insights for human-centered autonomous mobility.

The remainder of this paper is organized as follows. Section 2 describes the related work relevant to our proposal. Section 3 describes the system. Section 4 details the aspects of the human-centered approach followed during the design of the system. Section 5 describes the methodological aspects of the preliminary assessment conducted to validate the developed system. Section 6 discusses the results and limitations of this study. Finally, Section 7 presents the conclusions and future work.

## 2. Related Work

Autonomous driving systems and their control interfaces are a major driver of innovation in the transportation industry, accelerating the development of both autonomous navigation algorithms and new paradigms for remote supervision and control, with increasing emphasis on human-in-the-loop systems that ensure safety, accountability, and adaptability in uncertain environments. The SAE J3016 standard [3] provides a taxonomy of automation levels, laying the foundation for the integration of human oversight into vehicle control systems. Although fully autonomous driving remains a long-term goal, real-world deployment requires robust teleoperation frameworks to manage edge cases and unstructured scenarios.

Within this domain, Immersive technologies, such as Virtual Reality (VR) and Augmented Reality (AR), are gaining traction for their ability to enhance situational awareness and intuitive control. The virtuality continuum proposed by Milgram et al. [4] highlights the spectrum of immersive interfaces (which can range from simple data overlays to full sensory substitution) that enable operators to better perceive and act in remote or simulated environments, enhancing situational awareness and bridging the sensory gap between human operators and autonomous systems.

Head-Mounted Displays (HMDs) have shown promise for teleoperation. Previous work has extensively analyzed the challenges in the usage of HDMs with environments replicating control cabins virtually and allowing for embodied interaction with robotic or autonomous systems. For instance, Kalinov et al. [5] presented a virtual reality interface designed to interact with a robotic system for automated inventory management. The system allows operators to use an HMD to control a system based on unmanned ground and aerial vehicles. Similarly, Luo et al. [6] explored the first-person view operation of unmanned ground vehicles (UGV). Specifically, they studied the problems of perceiving distances suffered by operators using HMDs and VR. They proposed the use of on-vehicle sensors, vibro-actuators, and LED lights placed inside the HMD to provide distance information of obstacles around the UGV. Other examples include Whitney et al. [7], who introduced a VR interface to allow users to teleoperate a physical robot in real time, controlling their point of view in the scenes to increase situational awareness; Yoon et al. [8], who focused their research on the teleoperation of welding robots in construction and compared different dynamic viewpoint control techniques; Barentine et al. [9], who presented a VR-based robot remote control system together with a novel control algorithm; and Udekwe and Seyyedhasani [10], who described a VR system designed to teleoperate a robotic system for agricultural applications.

However, several studies have identified limitations in current VR-based teleoperation systems. Latency, jitter, and network performance continue to impact user experience and operational effectiveness [11]. This is especially problematic in safety-critical contexts, where delays in perception or control can compromise decision making [2]. Cognitive ergonomics is another critical concern. Poorly designed VR systems may increase the mental workload [12]. In particular, multimodal VR interfaces present overlapping or poorly prioritized stimuli, which may impair decision-making [13]. The mismatch between visual, auditory, and haptic feedback can increase cognitive load, leading to operator fatigue and reduced trust in the system [14]. Addressing this issue requires human-centered design approaches that prioritize clarity, hierarchy of information, and adaptive feedback mechanisms based on user needs and operational demands.

To overcome these challenges, recent efforts have focused on integrating advanced feedback modalities and leveraging simulation platforms for safe and repeatable tests. Previous works have shown that 3D simulators can assist in evaluating cooperative driving systems and testing human-machine interfaces (HMIs) in a controlled and repeatable manner [15]. These platforms offer valuable insights into driver behavior, communication protocols, and interface responsiveness before deployment in real-world scenarios. Such approaches align with current trends in teleoperation research, in which virtual environments serve as testbeds for refining control strategies, visualizations, and interaction paradigms.

Recent efforts have also leveraged middleware, such as the Robot Operating System (ROS) and game engines like Unity, to bridge physical and virtual systems and enable bi-directional communication between autonomous vehicles and immersive simulation environments [16,17]. The use of ROS-Unity or ROS-Isaac bridges enables closer synchronization between the robot middleware and immersive environments. These hybrid architectures facilitate the deployment of virtual testbeds and real-time control frameworks, thereby providing flexibility for system development and evaluation.

Moreover, multimodal feedback, which combines haptics, visuals, and sound, has been shown to improve spatial perception and reduce error rates in remote operations [6,14]. However, the trade-off between sensory richness and cognitive load remains an open research question. Studies on in-vehicle information systems and ergonomic assessments, such as IC-DEEP [18], have also contributed to refining human-centered design principles in vehicle control interfaces, highlighting the importance of usability testing and human factor analysis in the design of teleoperation interfaces.

Emerging research emphasizes the potential of 5G networks to reduce latency and support real-time remote operations [19,20], as well as the role of generative AI in streamlining sensory processing, adaptive feedback, and user assistance [21]. These technologies form the backbone of next-generation teleoperation platforms, combining low-latency infrastructure with predictive interaction models to improve system responsiveness and user trust.

In summary, while progress has been made in developing immersive teleoperation solutions, challenges remain in achieving sensory coherence, minimizing delays, and supporting intuitive multimodal interactions. This paper builds on this body of work by introducing a novel VR-based interface that integrates real-time simulation, gestural and physical controls, and multisensory feedback for the teleoperation of autonomous vehicles in emergency scenarios.

## 3. Description of the System

The proposed architecture was designed to enable immersive teleoperation of mobile robots in both simulated and real environments. It integrates advanced simulation technologies, robotic middleware, virtual reality user interfaces, generative AI, and haptic feedback. The modular approach facilitates a seamless transition between simulations and physical deployments, supporting multimodal control paradigms.

**Figure 1:**
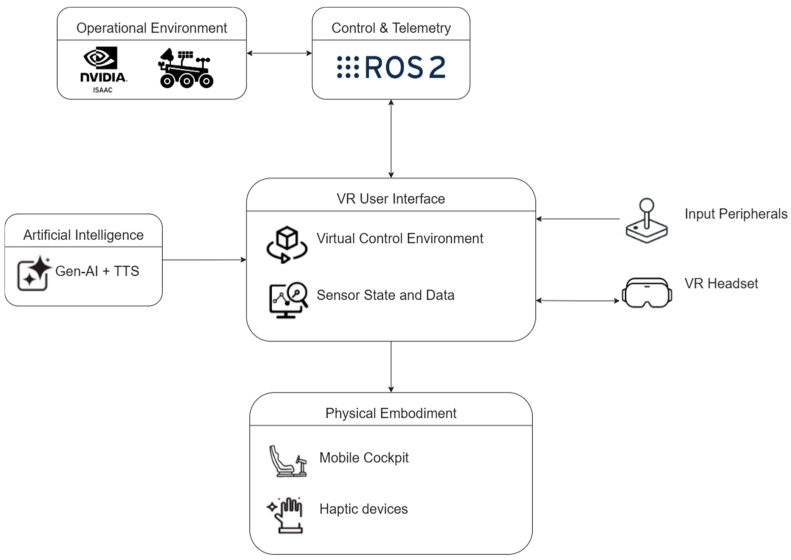
Modular architecture of an immersive teleoperation system.

The system, as shown in Figure 1, is structured into five layers: (i) the operational environment layer, which may consist of either a real or simulated robot; (ii) the control and telemetry layer, built on Robot Operating System 2 (ROS 2) [22] for communication tasks; (iii) the immersive VR user interface, which enables the operator to visualize and interact with the remote environment; (iv) the artificial intelligence layer, providing analysis and assistance capabilities through generative AI; and (v) the physical embodiment layer, designed to enhance user perception through multisensory stimuli. This layered architecture supports scalability, interoperability, and integration of new functionalities. The VR user interface integrates sensor data, control mechanisms, and AI-generated descriptions in a virtual environment. The operator interacts via a VR headset and input peripherals, while multisensory feedback is delivered through the physical embodiment layer, including a mobile cockpit and haptic devices.

The control and telemetry layers allow real-time communication across layers in the asynchronous mode. Furthermore, it permits communication with the operational environment, which can be either simulated (e.g., via NVIDIA Isaac Sim) or physical. It is based on ROS 2 as the distributed middleware that connects the sensors, actuators, and VR interface. Although its adoption varies across domains, ROS 2 is increasingly being adopted as a standard framework for programming and controlling robotic systems, particularly in research and development contexts. By abstracting communication through ROS 2, the immersive interface remains decoupled from the specific robotic platform, thereby enabling a high degree of modularity and reusability. This architectural decision facilitates the deployment of a unified software stack that operates consistently across both simulated environments (e.g., using NVIDIA Isaac Sim) and physical robotic systems without necessitating architectural modifications.

### 3.1. Operational Environment Layer

This layer constitutes the physical or simulated foundation on which the teleoperation system operates. It includes all hardware components involved in remote operation (sensors, actuators, mobile platforms, and embedded devices), whether deployed in a real-world setting or represented in a digital simulation. This layer can be composed of a physical robotic platform operating under real conditions or its simulated counterpart in a virtual environment, enabling the development, testing, and validation of functionalities without physical risk.

**Figure 2:**
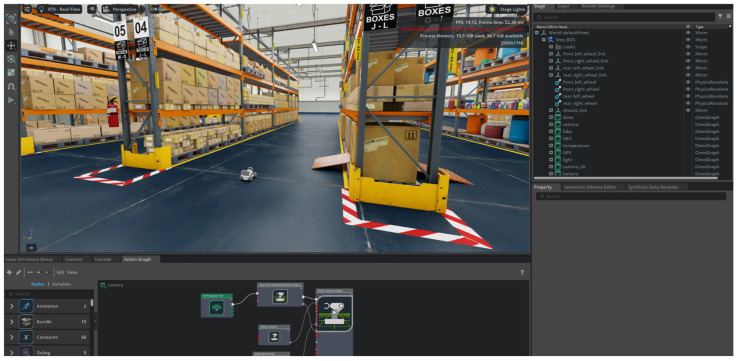
Virtual warehouse environment was developed in NVIDIA Isaac SIM, designed as a base scenario for system experimentation.

Throughout the development process, various experimental configurations were employed to test the immersive interface at different levels of complexity, such as an NVIDIA JetBot platform for early-stage prototyping, a custom-built robotic inspection platform for industrial use, and an advanced simulation in NVIDIA Isaac Sim using the AgileX LIMO model available in the official catalog. This simulated environment replicates an industrial warehouse inspection scenario (Figure 2), providing a controlled and repeatable setting for testing the system’s behavior and integration with the overall architecture via ROS 2.

This layer serves as the starting point for data acquisition, command execution, and performance evaluation in both the real and simulated operational contexts.

#### Simulated Robotic Platform

The Simulated Robotic Platform subsystem provides a high-fidelity digital twin environment for autonomous vehicle experimentation and validation. In this work, a simulation was implemented using NVIDIA Isaac Sim, a GPU-accelerated robotics simulator built on NVIDIA Omniverse [23]. In the context of Connected and Autonomous Vehicles, this layer enables full-stack simulation, including simulated sensor fusion, localization, mapping, navigation, and teleoperation. The digital twin accurately reflects the behavior of the physical robot, enabling fast transitions from simulation to reality with minimal reconfiguration.

The proposed system is not restricted to a specific robotic platform. While a specific robot from the Isaac Sim asset library was used in this study, the architecture is modular and adaptable to a broad range of mobile robots. Any robot equipped with a front-facing RGB camera, 360° LiDAR, and an IMU, and capable of publishing the required ROS 2 topics to the Control and Telemetry Layer can be integrated with minimal adaptation. This flexibility ensures applicability to real-world platforms commonly used in industrial and research settings and facilitates the transferability of the findings to diverse robotic configurations.

Moreover, the interchangeability between physical and simulated agents promotes hybrid deployment modes. For example, the operator can seamlessly switch from interacting with the robot in a simulation to controlling a real unit using the same interface and data structures, which is critical in high-risk or hard-to-access environments.

### 3.2. Control and Telemetry Layer

The Control and Telemetry Layer forms the foundational communication infrastructure of the system and is responsible for orchestrating real-time interactions between the robot (whether physical or simulated) and the surrounding ecosystem of interfaces and subsystems. At the core of this layer is ROS 2, an open-source middleware framework designed for modular and scalable robotics applications. ROS 2 provides essential communication primitives (topics, services, actions, and parameters) that enable distributed nodes to exchange information asynchronously and with temporal determinism. These nodes represent functional components, such as sensor interfaces (e.g., cameras, LiDAR, and IMU), control algorithms, localization systems, and interface modules. ROS 2 ensures real-time, low-latency data streaming through a Data Distribution Service (DDS)-based transport layer, which is crucial for robotic systems operating in dynamic and potentially hazardous environments. Table 1 presents the details of the messages and data handled by the system via ROS2 topics.

This architecture enables seamless integration between the robot’s physical and operational layers and external systems, ensuring consistent operation between the real robotic platform and its state representation within the control environment. By abstracting the hardware and simulation under a unified interface, the control and telemetry layer provides a hardware-agnostic interface for upper-layer functionalities, including immersive visualization and AI-based interpretation.

### 3.3. VR-Based User Interface Layer

This layer consists of a virtual reality environment developed in Unity, which receives and sends data via the ROS-TCP-Connector bridge [24]. At runtime, a ROS 2 “endpoint” node listens on a configurable TCP port and exposes a bidirectional channel for the message exchange. The ROSConnection component of Unity initiates a TCP handshake with this endpoint, effectively registering the VR application as a ROS 2 node. Once connected, the endpoint serializes and deserializes standard ROS 2 messages and custom status topics—into a compact byte stream for transport over TCP/IP.

**Figure 3:**
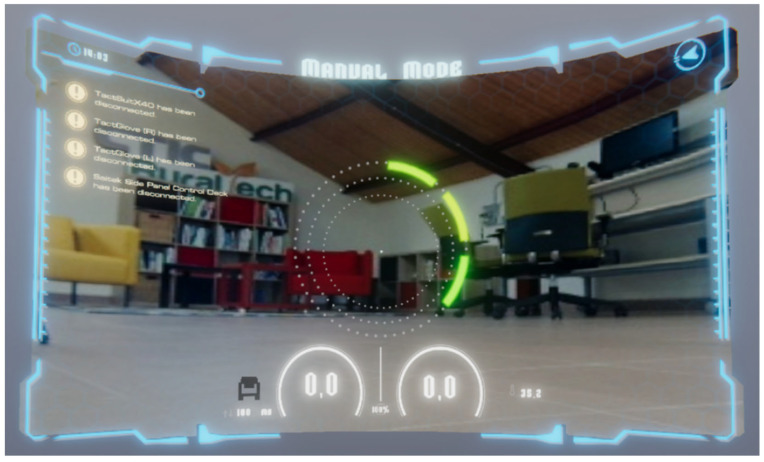
Control panel of the system within a Virtual Reality environment.

The design enables both visualization and remote control by operators, rendering a custom 3D monitoring environment in the VR format. The example in Figure 3 shows a real-world scene captured during early-stage prototyping using the NVIDIA JetBot platform. The interface was designed to enhance the operator’s experience through an intuitive, manipulable, and real-time representation of the robot’s state and surroundings. It acts as the human-facing layer of the architecture, while the definition of how interaction and feedback are structured to reduce cognitive load and facilitate effective decision-making in dynamic scenarios is covered in the following sections.

By abstracting all communication through the ROS-TCP-Connector, the VR interface remains agnostic to whether the data originates from a physical robot or its Isaac Sim counterpart. This design ensures that the Unity scene consistently reflects real-time robot behavior and that operator commands are relayed with minimal latency, thereby enhancing immersion and decision-making in dynamic teleoperation scenarios.

### 3.4. Artificial Intelligence Layer

The system incorporates a dedicated artificial intelligence module for processing visual information captured by the robotic platform, which is directly integrated with the immersive Virtual Reality interface. Upon user request, the front camera image was analyzed using a multimodal generative model deployed on in-house servers equipped with GPU-accelerated computing capacity. The model employed is an instance of Mistral Small 3.1, which is designed for visual-linguistic understanding tasks. The output of the analysis is a natural language description presented both visually (as overlaid text within the virtual environment) and audibly (via speech synthesis), enhancing the operator’s situational awareness and providing multimodal feedback. By deploying on-premises AI models, we ensure low latency and preserve the privacy of sensitive operational data.

Thus, this layer transforms raw visual data into semantically enriched information through two main subsystems:

- Multimodal Generative Inference: The current camera frame is sent via an HTTP request to the generative AI service. The Mistral Small 3.1 model [25] encodes images using a visual backbone network, producing a dense representation interpreted by the language decoder. The output is an automatic natural language description (text) summarizing the most relevant scene elements, including the detected objects, spatial relationships, and relevant context.
- Text-to-Speech Conversion: The description generated by the previous module is forwarded to an in-house speech synthesis node running Whisper v3 Large [26], which is a neural vocoding model optimized for high-fidelity voice generation. This subsystem converts the text into a mel spectrogram using the model’s acoustic encoder and subsequently applies the vocoder to produce a 16-bit, 16 kHz PCM audio signal.

Both outputs, text and audio, are delivered to the Virtual Reality user interface, where they are integrated into the control environment to provide a multisensory representation of the remote scene, thereby facilitating operator interpretation in dynamic or high-uncertainty contexts. This functionality enhances accessibility, particularly for users with sensory limitations or in low-perception environments, and provides decision support by offering concise and high-level interpretations of the remote scene.

### 3.5. Haptic and Physical Feedback Layer

This layer incorporates tactile feedback devices in addition to a motion platform. Although these devices are connected to the VR interface, they are integrated at the system level to provide sensory cues that are synchronized with the robot’s environment. The purpose of this layer is to enhance the realism and intuitiveness of the interaction by delivering sensory responses aligned with events detected in the operational environment, thereby improving the user’s perception during remote operation.

Specifically, we employed a motion platform that tilted the cockpit with two degrees of freedom (2DOF), adjusting it according to the inclination measured by the robot’s IMU in the operational layer. Additionally, a haptic vest and gloves deliver vibrations at various points on the user’s body with different intensities. These vibrations are linked to proximity measurements: the vibration intensity increases as the user approaches an obstacle, and the vibration location on the body corresponds to the direction of approach. Furthermore, a vibration pad installed on the seat reproduces the rattling of the remote machine, which is connected via an audio channel. We selected these types of vibro-actuators and decided not to produce vibrations in the head-mounted display because of probable discomfort, as reported in a previous work [6].

The effects of these devices and feedback cues on user experience are detailed in the following section.

## 4. Human-Centered Design

The design of the immersive interface is based on principles inspired by the ISO 9241-210 standard [27]. This standard defines human-centered design as “an approach to systems design and development that aims to make interactive systems more usable by focusing on the use of the system and applying human factors/ergonomics and usability knowledge and techniques.” Thus, to ensure that the solution meets the real needs of operators, the design process was conducted through co-creation with experienced industrial machinery operators. This collaborative approach allowed the identification and prioritization of specific requirements for remote teleoperation and adaptation of the interface to facilitate intuitive and efficient interaction in complex and dynamic environments. The goal is to optimize the user experience, improve safety, and reduce operator fatigue during operation, ensuring that the technology supports and enhances human capabilities in remote equipment control.

**Figure 4:**
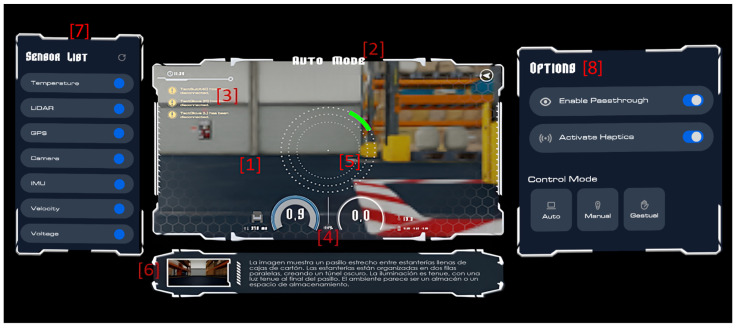
Components of the human-centered interface of the virtual control environment (numbering from previous list).

The main components of the visual interface in the virtual control environment shown in Figure 4 (numbered and referenced) are as follows:

1. Live video streaming from the front-facing camera mounted on the robot provides a first-person perspective of the operational environment.
2. The border around the control panel indicates the current operation mode of the robotic platform through text and color variation. There are three main modes:
  - Autonomous control, where the vehicle navigates autonomously using its robotic programming.
  - The emergency mode completely stops the vehicle’s movement. The operator can activate this mode on demand when an abnormal or hazardous situation is detected during autonomous driving supervision.
  - Manual control allows the user to teleoperate the vehicle via an immersive interface, either through physical peripherals or gesture commands.
3. In the top-left corner, a notification panel displays real-time alerts, the connection status of key devices, and other critical errors.
4. At the bottom of the control panel, various informational widgets regarding the vehicle’s current status are grouped. The most relevant are the radial bars, which indicate the robot’s instantaneous linear and angular speeds. The remaining battery charge of the robotic platform is shown, and to its right are the robot’s internal temperature and GPS coordinates. On the left, a schematic representation of the robot shows the inclination measured by the vehicle’s IMU in the operational environment, which is also translated to the motion platform that tilts the cockpit along two axes.
5. At the center of the control panel, a 360° proximity radar displays the closeness of environmental elements using concentric rings. This radar represents obstacle distances based on LIDAR data, employing a color code ranging from green (safe distance) to red (critical proximity). This feedback is multisensorially reinforced with directional haptic feedback provided by the gloves and vest, producing vibrations in the corresponding directions.
6. Below the control panel is the system communication panel, which activates contextually only when necessary. It overlays informational text for the user, such as the environment description requested from the Artificial Intelligence layer or instructions for familiarization with the interface. In addition to displaying the text, narration is played, created using the text-to-speech model.
7. The sensor list located to the left of the control panel allows the user to activate or deactivate the connection to the physical sensors in the operational environment layer at any time, and adjust the received information according to their preferences.
8. The options panel to the right of the control panel allows the user to adjust some features of the virtual environment according to their preferences (e.g., disabling haptic feedback). It also allows switching between different remote-vehicle control modes.

### 4.1. Ergonomics and Multisensory Feedback

The proposed immersive teleoperation system integrates a multimodal feedback architecture designed to support a wide range of use cases. Rather than targeting a single application domain, the system is conceived as a generic and adaptable framework in which visual, auditory, haptic, and physical stimuli can be selectively activated and configured depending on the operational context, embedded hardware of the robotic platform used, and task complexity. This approach facilitates the operator’s situational awareness while mitigating cognitive overload and sensory desynchronization in immersive environments.

- Visual Feedback: The immersive interface presents the robot’s environment, camera streams, system diagnostic data, and warning signals in real time. Representation in a virtual environment facilitates spatial perception and allows the operator to maintain an overview of the operating context.
- Auditory Feedback: Audio cues can include both synthetic speech (e.g., system instructions or messages generated by the Artificial Intelligence Layer) and real-time environmental audio captured by the robot. The auditory layer can be extended depending on the perceptual demands of a scenario.
- Haptic Feedback: The system supports integration with haptic gloves and vests to convey spatial information via vibrations. These devices are capable of delivering directional feedback with adjustable intensity, which was initially proposed to convey proximity-based information about the operational environment, although their use can be tailored or omitted according to task requirements.
- Physical Feedback: The system can interface with motion platforms and vibrotactile seat pads to simulate movement and mechanical feedback. These components close the loop between kinesthetic and visual inputs, enhancing the realism of the experience as required.
- Adaptive ergonomics: The physical cockpit is adjustable to suit the operator’s physical characteristics. Similarly, the VR control interface allows the layout of the control panels to be modified, offering customizable configurations aimed at improving ergonomics and reducing the cognitive load.

Together, these mechanisms favor the user’s sense of presence and bodily integration into the scene perceived through a virtual environment. At the same time, it helps to reduce sensory disorientation and fatigue while improving operational effectiveness in dynamic or critical environments.

**Figure 5:**
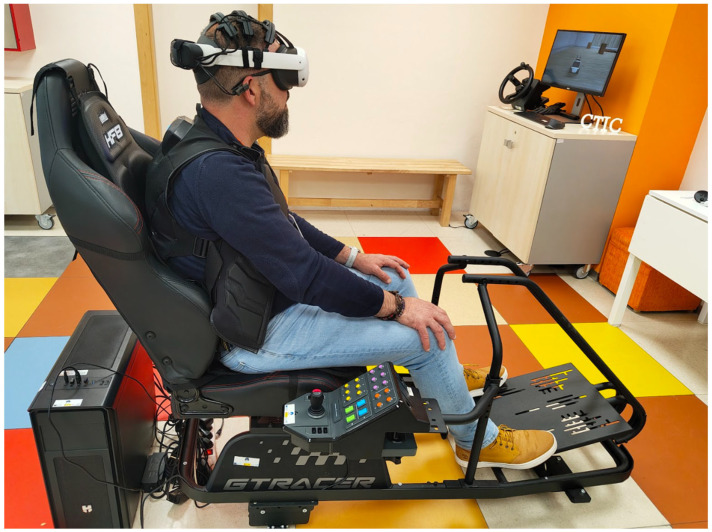
Complete experimental setup showing a fully equipped operator seated in the immersive cockpit.

An example of a complete experimental setup is illustrated in Figure 5, where a user is shown seated in an immersive cockpit with all core wearable and VR devices integrated. This configuration includes a VR headset, haptic vest, vibrotactile pad, and motion platform that applies physical movement.

### 4.2. Interaction Modalities: Physical and Gesture-Based Controls

The system implements two main modalities for robot teleoperation: physical peripheral input and gesture-based recognition. Both options can be activated at any time, allowing the operator to choose the most suitable method based on their personal preference or the operational context.

**Figure 6:**
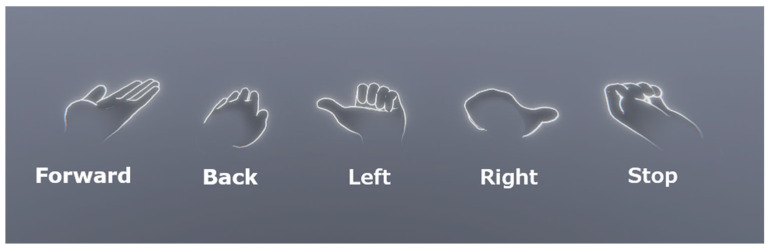
Main hand positions for robot control in gesture-based teleoperation. From left to right, the gestures are to move forward, move backward, turn left, turn right, and stop.

**Figure 7:**
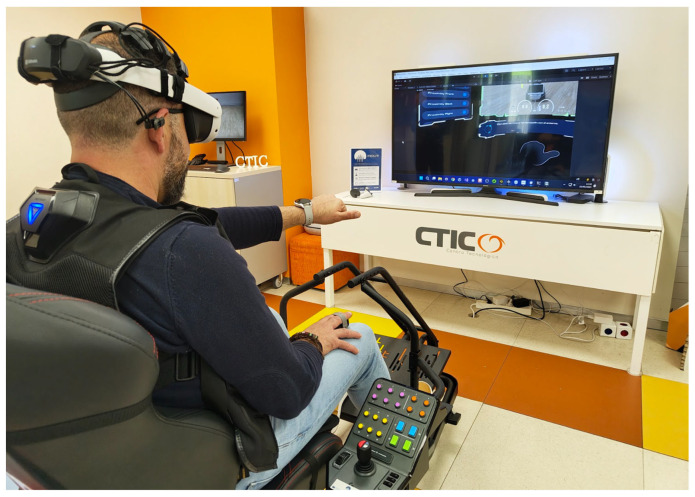
Operator controlling a robot using gestures: the robot should turn right.

- Interaction via physical controls: The user is provided with a physical control panel equipped with buttons and a joystick to trigger emergency stops and control the robot’s motion (Figure 5). This physical panel has a precisely aligned digital replica in the virtual environment. Through the passthrough mode in the VR headset, the virtual panel is spatially calibrated to visually overlap with the real hardware, enabling natural and accurate interaction with physical elements while remaining immersed in VR.
- Interaction via gesture commands: Leveraging the hand-tracking capabilities of the Meta Quest 3 headset and its Unity integration, the system supports real-time gesture recognition without requiring physical controllers. This modality allows the user to navigate interface menus and issue remote movement commands to the robot using a set of predefined hand gestures. These gestures, shown in Figure 6, were carefully designed to be unambiguous and intuitive and were presented as part of the onboarding process (Figure 7).

## 5. Evaluation of the System

### 5.1. Population

Participants were selected through a convenience sampling approach and consisted of CTIC researchers, none of whom were directly or indirectly involved in the design or development of the experiment. Before the experiment, the participants completed a pre-study questionnaire that captured demographic data and baseline attitudes toward autonomous systems. All participants provided informed consent and were briefed on the goals and procedures of the study.

A total of 15 participants were involved in the evaluation sessions; the gender distribution was balanced, with 53% identifying as male and 47% as female, as well as age groups, with 47% under and 53% over 30 years old.

Regarding technological background, 87% of participants reported prior experience with virtual reality systems, and 47% had occasionally used driving simulators. Regarding driving skills, only two participants had no prior experience operating vehicles (real or simulated), while the remaining sample rated their driving experience as moderate (two participants) or high (11 participants).

Familiarity with autonomous systems was limited; only one participant had ever ridden an autonomous vehicle. However, a larger proportion (10 participants) had previously interacted with autonomous technologies as occasional users.

The participants did not anticipate a significant variation in their confidence levels when using the immersive interface prior to the experiment. The average baseline confidence was 3.13 out of 5, while the expected confidence when using the interface increased slightly to 3.33.

This distribution ensured a heterogeneous sample combining diverse levels of expertise and familiarity with immersive and autonomous systems while preserving a sufficient baseline of user competence for meaningful task execution and assessment.

### 5.2. Test Scenarios

**Figure 8:**
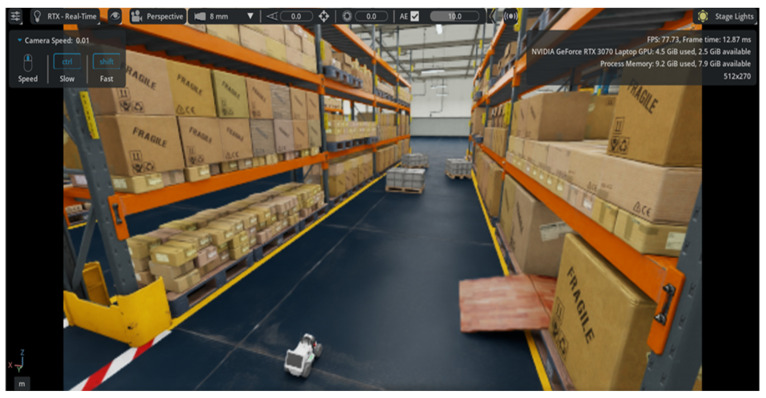
Scene generated in NVIDIA Isaac Sim, depicting a warehouse where an autonomous robot performs inspection routes.

The experimental setup was designed for a representative use case involving autonomous supervision in an industrial warehouse environment. To ensure safety and eliminate any risk of accidents during the study, no physical robotic platform was employed. All test scenarios were conducted using a simulated robot within the NVIDIA Isaac Sim environment, as shown in Figure 8.

Specifically, the LIMO model from AgileX Robotics, a four-wheeled robotic platform used in real-world scenarios and included in Isaac Sim’s default asset library, was selected due to its sensor configuration and morphology, which are easily transferable to a wide range of autonomous mobile robots used in industrial or inspection contexts. A simulated 360° LiDAR sensor was integrated into the robot to provide proximity detection capabilities.

The participants were exposed to controlled emergency situations during the autonomous operation of the robot along a predefined route within the simulated warehouse. In certain scenarios, obstacles were introduced along the paths. If no intervention was performed, the robot inevitably collided with them. The participant was responsible for supervising the robot and taking control when necessary through the immersive interface.

Four different tasks were included in the evaluation, with an estimated completion time of 30 min. The first (T01) served as a familiarization stage, guiding the participants through the immersive environment and interface elements. Instructions were delivered via text and speech, explaining the meanings of the different alerts, visual cues, and interaction methods. It was structured into five sequential phases to ensure that users acquired a basic understanding of the system, regardless of prior experience, before engaging in autonomous supervision tasks.

- System Introduction: Participants received an overview of the robot, its virtual environment, and the various communication systems integrated into the immersive interface. Special emphasis was placed on the role of proximity feedback in obstacle detection and collision warning.
- Calibration Phase: A manual alignment procedure was conducted to calibrate the physical button panel with its virtual replica in the VR environment to ensure spatial congruence through a passthrough overlay.
- Emergency Scenario Demonstration: Participants were exposed to a simulated collision scenario to demonstrate the system’s multimodal warning mechanisms. A virtual obstacle was introduced into the predefined trajectory of the robot, triggering a collision warning. The participants were prompted to observe the visual and vibrotactile feedback presented by the immersive interface and then stop the robot’s movement by activating the emergency mode.
- Manual Control Familiarization: Participants were guided through the fundamental mechanics of controlling the robot using a physical joystick and button panel.
- Gesture-Based Control Familiarization: Participants were introduced to the gesture recognition system powered by hand-tracking capabilities. A predefined set of gestures was taught for remote robot intervention.

The remaining three tasks (T02–T04) required participants to independently supervise the robot and intervene at their discretion based on what they had learned in the familiarization phase. The execution order of these tasks was randomized across participants to avoid learning effects and to mitigate behavioral biases. One scenario presented a flawless route with no anomalies and required no intervention (T02). The other two tasks introduced critical obstacles into the robot’s trajectory, creating emergency situations in which a collision would occur unless the user actively overrode the autonomous control. Upon perceiving the warning combination of signals (particularly, the alerting pattern in the 360° proximity radar and the high-intensity directional vibration from the haptic vest), participants were expected to intervene to prevent the collision by switching to emergency mode and stopping the autonomous movement. They then manually teleoperated the robot using either the physical control panel (T03) or hand-gesture commands (T04) to safely navigate around the obstacle and resume the task.

### 5.3. Configuration of Multisensory Feedback Mechanisms

The feedback architecture of the system described in Section 4.1 was adapted and fine-tuned for the teleoperation tasks to be performed during the experiments, taking into account the selected LIMO robotic platform. In particular, the following elements were used:

- Haptic Feedback:
  - TactGlove DK2 [28] gloves: Vibrations were triggered on the entire hand whenever the robot approached a predefined safety threshold near obstacles. The hand corresponding to the direction of the threat (left or right) was enabled. The vibration intensity was scaled proportionally to the proximity distance, with a maximum intensity of 25 cm. Although these gloves were fully integrated and configured as part of the system, they were intentionally excluded from the experiments to prevent interference with data collection for a concurrent study.
  - TactSuit X40 [29] vest: Directional vibrations were activated upon the detection of objects within a 1-m radius around the robot. The vibrations were continuous, and their intensity was scaled proportionally to the proximity distance, with a maximum intensity at 25 cm. Vibrations were mapped in the direction of the approaching obstacle (front or back). Together with the gloves, these elements allow spatial awareness to be maintained without the need to divert visual attention.
  - HF8 seat pad [30]: This component transformed the audio signal into vibration within the cockpit seat itself, providing continuous tactile feedback that emulates the robot’s mechanical resonance during movement.
- Auditory Feedback:
  - System instructions: During the familiarization stage, instructions and messages were presented to the user via synthesized speech to reinforce visual prompts.
  - Environmental audio: Real-time transmission of the robot’s microphone was emulated, allowing the participants to hear motor sounds and ambient noise from the operational environment.
- Physical Feedback:
  - Motion Plus [31] motion platform: This element produces pitch and roll movements (2DOF) on the entire cockpit based on the inertial data from the robot (IMU).

### 5.4. Evaluation Metrics

The evaluation protocol integrated both objective and subjective data collection methods to assess the system’s performance and user behavior across the experimental scenarios.

Objective metrics were automatically logged by the system during each task execution. The following indicators were recorded for each participant:

- Participant ID: An anonymized code that allows the experiment to be matched to the participant’s subjective data.
- Task Identifier: Scenarios executed, ranging from T01 to T04.
- Task Outcome: Final state of task execution, categorized as follows:
  - Shock: The robot collided with an obstacle, as detected by the LiDAR sensor (threshold: 25 cm). This state overrides all other outcomes.
  - False Positive: The user took control in a scenario with no emergency condition by design (T02).
  - Preemptive: The user intervened before the emergency event occurred in a scenario that included a potential collision (T03, T04).
  - Reaction: An emergency condition occurred, and the user intervened before an actual collision occurred (T03, T04).
  - No Intervention: The user did not intervene during task execution.
- Emergency Event Timestamp: Time log of when the LiDAR system registered an alert state (75 cm distance threshold).
- User Intervention Timestamp: Time log of when the user entered the emergency mode to stop the autonomous motion.
- Input Mode: Control method used during the intervention, either physical (button panel) or gestural (hand-tracking).
- Success: A binary indicator reflecting whether the task was completed without collisions (i.e., any outcome other than “Shock”).

In addition to these system logs, subjective metrics were collected using two structured questionnaires and direct observation.

The pre-experiment questionnaire (PRE) focused on capturing participants’ demographic data and their initial expectations of the immersive interface. It included self-assessed levels of prior experience with VR and driving simulators, familiarity with autonomous systems, and anticipated trust in the system, using a 5-point Likert scale.

The post-experiment questionnaire (POST) was designed to gather feedback on the interaction experience, perceived usability, and participants’ workload. It was conducted after executing the four tasks of the experiment, and assessed trust variation after the use of the system, perceived workload following the NASA-TLX dimensions (mental demand, physical demand, temporal demand, performance, effort, and frustration) [32], system usability for interface evaluation, and presence and embodiment.

Additionally, an observational checklist was completed by the research team during each session to record behavioral and procedural indicators, such as the observed time to intervene in emergency tasks, hesitation or confusion during interaction, preference or ease of use observed for one input modality over the other, and physical signs of fatigue or discomfort (e.g., repositioning or removing the headset).

Together, these metrics provide a multi-dimensional picture of task performance, system usability, and user experience within immersive teleoperation scenarios. These self-reported measures were analyzed to complement behavioral data metrics and provide insights into how interface design affects cognitive processing and trust formation.

## 6. Results and Discussion

This section presents an initial analysis of the data collected during the user trials, focusing on the behavioral performance and subjective feedback. Although the sample size is limited and the study is still ongoing, the early trends offer insights into the effectiveness of the immersive interface and the different interaction modalities tested.

### 6.1. User Expectations and Initial Attitudes

Prior to engaging with the immersive teleoperation system, participants were invited to reflect on their expectations and potential concerns to help contextualize later behavioral outcomes and subjective responses.

When asked about anticipated perceived difficulties in remote supervision and control, participants’ open responses revealed five main thematic clusters:

- Precision in remote control was the most frequently cited concern (five participants), with users expressing doubt about their ability to execute accurate or complex movements from a distance.
- Latency and delays were mentioned by three participants, who feared that response times might not be adequate in emergency situations.
- Spatial perception and scene complexity were also of concern to three individuals, particularly in relation to judging distances or interpreting a cluttered remote environment.
- Signal and alert interpretation were noted by two participants who anticipated difficulty in understanding system notifications or environmental cues.
- Vague or undefined concerns were expressed by three other participants, who provided responses that were not clearly tied to any specific functionality or system component.

In terms of what users expected to perceive via the immersive interface, responses were concentrated in three areas:

- Visual understanding of the environment was the most common expectation (13 participants), confirming a strong association between VR and environmental awareness.
- The sensation of movement (12 participants) and obstacle proximity perception (11 participants) followed closely, indicating that most users anticipated not only visual immersion but also kinesthetic or spatial feedback. One participant mentioned “real immersion” as a more holistic expectation, suggesting a broader engagement with the virtual scenario beyond specific sensory cues.

Finally, regarding uncertainties prior to the trial, most participants (nine) reported no major concerns. Among those who expressed reservations:

- VR-related limitations were cited by two individuals who referred to visual discomfort or previous negative experiences.
- System control apprehension (2 participants) pointed to doubts about effectively operating the robot in emergency situations.
- Personal physical or cognitive states (two participants) were identified as factors that might influence performance.
- One participant specifically mentioned latency-related concerns.

Overall, the responses indicate that most participants approached the system with a positive or neutral attitude, although a significant subset anticipated challenges related to control precision, system responsiveness, and spatial interpretation, which are the three aspects that the immersive interface is precisely designed to mitigate.

### 6.2. Perception of the Experience

**Figure 9:**
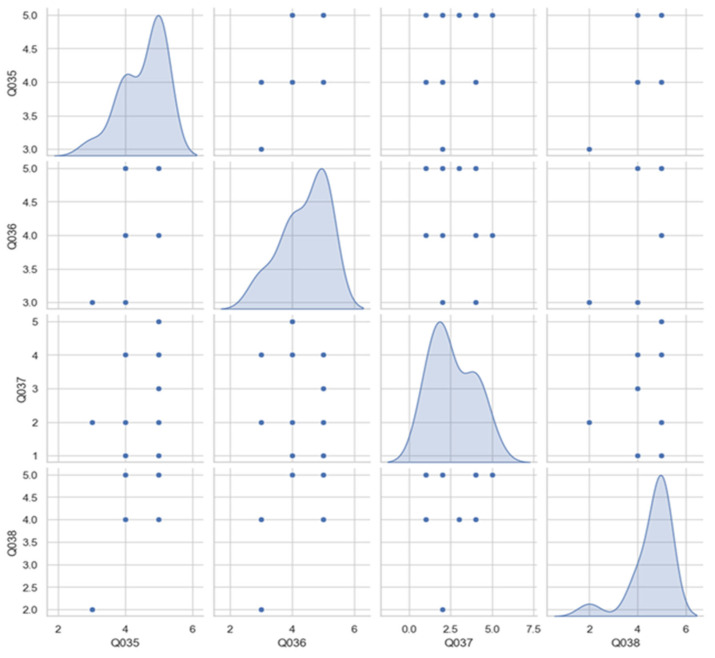
Immersion and presence indicators reported by the test participants and their correlations. The diagonal alignment of the scatter plot distributions indicates the presence of a correlation between the variables. Q035—Presence in a virtual environment. Q036—Natural interaction with the system. Q037—Immersion affected by latency (negative value). Q038—Sensory realism.

Participants’ subjective experience of immersion and presence within the virtual environment was assessed using four Likert-scale items in the post-experiment questionnaire (Q035–Q038). These items targeted the key dimensions of presence, natural interaction, sensory realism, and the impact of system latency. The correlation between these indicators was analyzed, as shown in Figure 9, revealing a strong relationship between the sense of presence in the virtual environment (Q035) and perceived sensory realism (Q038), with a correlation coefficient of 0.7. Additionally, a moderate correlation (0.58) was observed between the sense of presence (Q035) and perceived naturalness of interaction with the system (Q036).

The results indicate a strong overall sense of presence and realism. Participants felt physically present in the virtual environment (Q035) with a high average score of 4.53 out of 5, suggesting effective spatial and perceptual integration. They also reported being able to interact naturally with the system (Q036), with an average score of 4.4, highlighting the effectiveness of both the physical and gestural control mechanisms. Sensory realism (Q038), including visual, auditory, and haptic stimuli, received the highest average rating of 4.6, reflecting the successful multimodal integration of feedback cues designed to support situational awareness.

However, when asked whether latency affected their immersion experience (Q037), participants provided an average rating of 2.6, indicating that delays in system responsiveness were perceived and impactful. This suggests that while the interface was generally effective in conveying presence and supporting intuitive interaction, further optimization of responsiveness would enhance the overall immersive experience.

### 6.3. Comprehensibility of Information and Effectiveness in Control and Decision-Making

The system was positively evaluated in terms of how well it supported users’ understanding of the remote environment and their ability to take informed action. When asked whether the system responded adequately to their actions, 14 out of 15 participants responded affirmatively, while only one participant reported inconsistent feedback, indicating a high level of perceived responsiveness in the teleoperation interface.

Regarding decision-making clarity, the participants were asked whether they ever doubted the necessity of an intervention during the task. A large majority (12 participants) expressed no doubts and reported full confidence in their interpretation of the situation and control input. Among the remaining participants, two expressed uncertainty regarding whether the robot would autonomously avoid the obstacle, and one described momentary doubt due to difficulty interpreting the visual scene. These responses suggest that the information provided was generally clear and well-structured, allowing the participants to make prompt and accurate decisions in real time.

**Figure 10:**
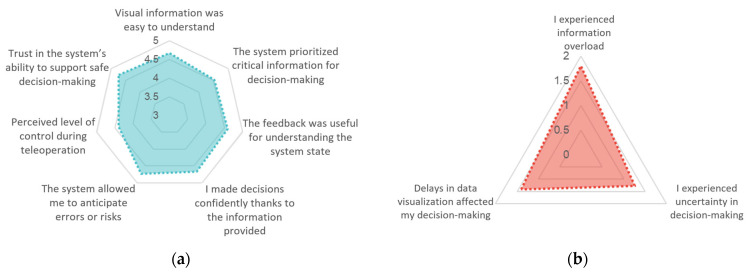
Mean values of quantitative assessments for positive (**a**) and negative (**b**) feedback items.

Quantitative evaluations further support this trend (Figure 10). Users were asked to rate the system in terms of usefulness, interpretability, and perceived control using a 1–5 Likert scale. The participants assigned an average score of 4.62 out of 5 to positive items, indicating a strong sense of control and confidence during teleoperation. In contrast, potentially negative experiences, such as information overload, uncertainty in decision-making, or delays in data rendering, were rated below 1.5 on average, suggesting that such issues were rare or negligible. These results highlight the efficacy of interface design in prioritizing relevant cues, minimizing cognitive load, and enabling decisive intervention when necessary.

### 6.4. Feedback Evaluation

**Figure 11:**
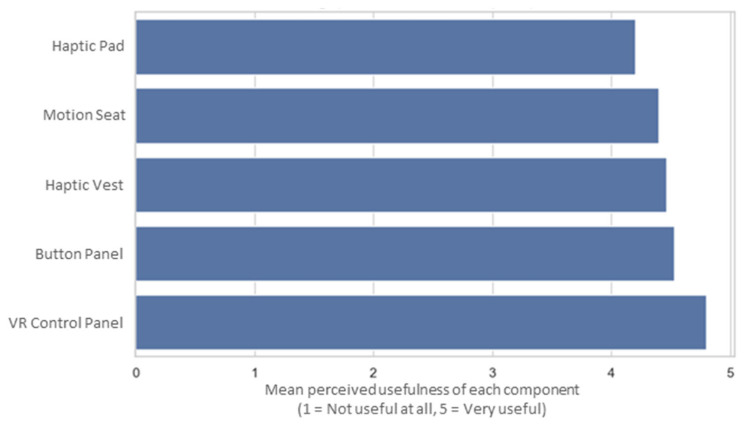
Average perceived usefulness per feedback device type on a 1–5 Likert scale.

All feedback components (visual, auditory, and haptic) were generally well received, with average scores equal to or above 4 out of 5, as shown in Figure 11, indicating a high level of perceived usefulness. Among them, the VR control panel was identified as the most valued element, providing intuitive and immediate access to system indicators and commands. In contrast, the haptic seat pad and tilting seat platform were perceived as less impactful, although they were still positively rated.

When asked whether any feedback elements were annoying, confusing, or unnecessary, most (nine participants) indicated that all components were helpful. Some isolated mentions included the intensity or duration of the vibro-tactile feedback (3 participants), as well as minor individual discomforts, such as difficulty accessing the emergency button, mild motion sickness, or perceived redundancy of some visual data. More details about this subjective assessment are as follows:

- Nine did not report any issues with feedback elements.
- Three participants found the haptic feedback to be occasionally uncomfortable or excessive (i.e., seat or vest vibration).
- Two participants reported inconveniences related to the physical interface (e.g., “The emergency button was too close to the accelerate/reverse button”, and “Looking away from the screen to see the button panel caused slight dizziness”).
- One participant mentioned a potential overload of visual information, though it was defined as not bothersome but rather unnecessary.

In response to the open-ended question about additional alerts or indicators that could improve awareness, six participants reported no need for enhancements and expressed satisfaction with the current setup. However, five participants suggested more varied auditory alerts to enhance environmental awareness. Three others proposed improvements to visual feedback, such as displaying the projected path of the robot. The suggestions for new feedback indicators may be grouped as follows:

- Six participants were fully satisfied with the current feedback mechanisms without suggesting any additions.
- Five participants recommended enhancing auditory feedback to improve awareness of the robot’s status or its proximity to obstacles.
- Three participants suggested the inclusion of additional visual mechanisms to support spatial awareness and provide more detailed information about the robot’s condition.
- One participant identified an interface interaction detail that could be improved to make the controls more intuitive, although this comment was not directly related to the specific question about alerts and indicators.

Regarding suggestions to enhance trust and coordination in complex situations, most participants expressed satisfaction with the current system and did not state a need for additional enhancements. Responses may be grouped as follows:

- No changes needed (satisfaction with the current system): eight participants.
- Improvements to auditory or multisensory communication: four participants.
- Enhancements in gesture interaction: Two participants.
- Improved visual awareness (e.g., rear camera): one participant.

These results show clear overall satisfaction with the system’s current feedback design, which participants found to be clear, useful, and non-overwhelming, even under demanding conditions. Nevertheless, a minority of users pointed out potential refinements to the system. The following main refinements are suggested:

- Adding more natural and context-sensitive audio messages, such as verbal proximity alerts, is also recommended.
- Improving haptic feedback modulation, especially by reducing the intensity or duration of prolonged alerts.
- Supporting gesture-based interactions with on-screen reminders or cues.
- Adding a rear-facing camera to enhance spatial awareness and operator confidence.

### 6.5. Ergonomics and Interaction

Regarding physical comfort, 73% of the participants reported that the system was comfortable to use, while the remaining 27% indicated that it was partially comfortable, with no negative responses. This reflects a generally positive assessment of the ergonomics of the system.

**Figure 12:**
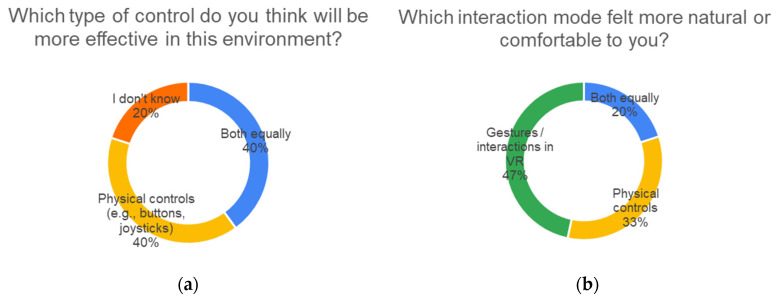
Comparison of control type preferences before (**a**) and after (**b**) the experiment.

Concerning user preferences between control modes, there was a notable shift between pre-(PRE) and post-experience evaluations (POST). As shown in Figure 12a, prior to the test, none of the participants expected gesture-based controls to be the most effective. Instead, 40% preferred physical controls, and another 40% anticipated liking both equally. However, Figure 12b shows that after the experiments, 47% of the participants preferred gesture-based interactions in VR, compared to 33% who favored physical controls and 20% who rated both equally.

Although most users (11 out of 15) did not report technical or usability issues, a relevant subset (four participants) identified challenges specifically linked to gesture control. These include technical limitations (such as the need for exaggerated movements or inconsistent gesture recognition) and cognitive difficulties, such as remembering gesture-action mappings.

**Figure 13:**
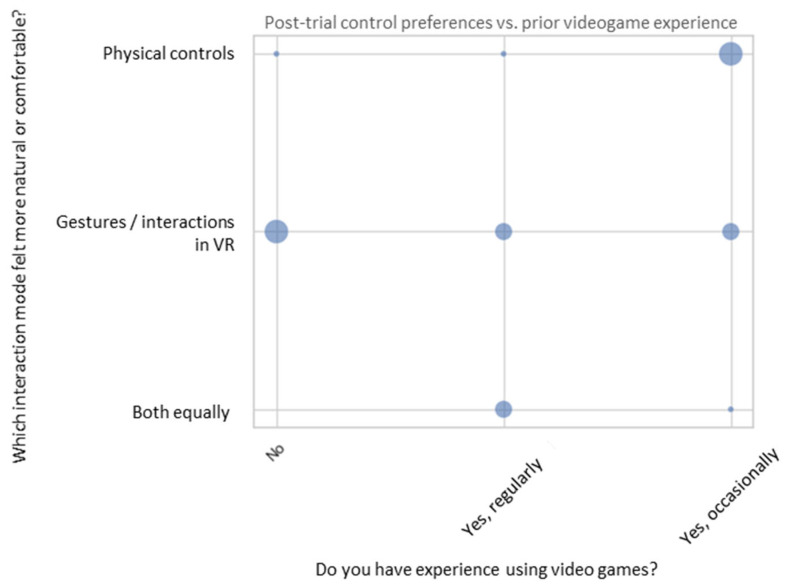
Post-trial preference for the most natural/comfortable type of control, segmented by the experience of participants with video games. Larger dot sizes represent a greater number of responses within those categories.

When analyzing control preferences in relation to previous gaming experience (as self-declared in the PRE questionnaire), an interesting pattern emerged, as shown in Figure 13: participants with “occasional” gaming experience tended to prefer physical controls, while participants with no gaming experience favored gesture-based VR interactions.

This suggests that a technological background may influence the perception of naturalness and usability. Experienced gamers are often more familiar with physical input devices (e.g., gamepads, keyboards) and may find them more precise or reliable. In contrast, participants without this background may perceive gesture-based control as more intuitive and direct, unburdened by their ingrained interaction habits.

This has relevant implications for the design of remote operation systems:

- Gesture-based interaction may be especially accessible to non-specialized users, such as industrial trainees, citizens interacting with public robotics systems, or first-time remote operators in emergency contexts.
- At the same time, a universal control scheme may not be feasible due to varied user backgrounds. Therefore, flexibility and adaptability in interface design are critical.

The system tested in this study already supports interchangeable control modes, allowing operators to switch between physical and gestural inputs based on their preferences. This adaptability can enhance both the user experience and operational effectiveness, particularly in real-world applications with diverse operator profiles.

### 6.6. Mental Workload

**Figure 14:**
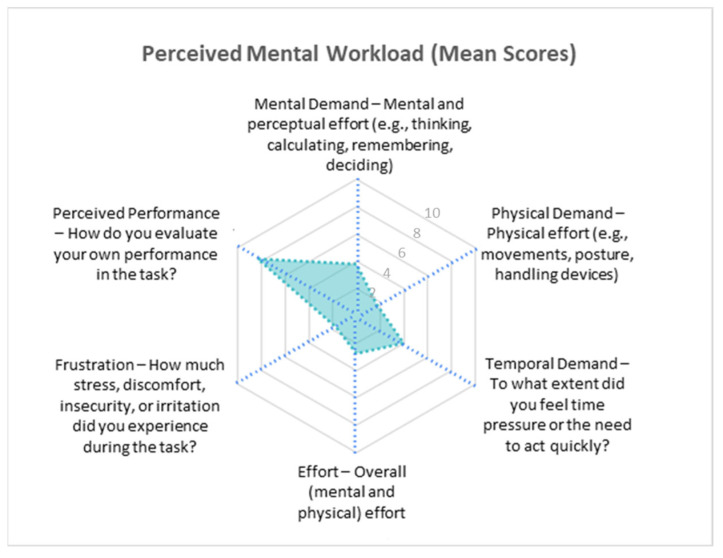
Mean scores of perceived mental workload dimensions in NASA-TLX test.

The evaluation results indicate that teleoperation using the proposed system was generally perceived as a cognitively accessible and physically comfortable experience. Figure 14 shows the mean perceived mental workload scores for each NASA-TLX dimension. Participants reported moderate mental demand (mean = 3.8/10) and time pressure (3.93/10), but low physical demand (1.67/10) and light overall effort (2.73/10). Most users found the task manageable, and frustration remained low (1.73/10), suggesting a fluid and comprehensible experience. The high self-rated performance (8.33/10) confirms that the participants largely felt competent and in control during the task. Nevertheless, the variability observed across all dimensions (particularly mental demand, with a standard deviation of 2.21) revealed individual differences in how the system was experienced. These variations highlight the importance of designing systems for diverse user profiles, especially in contexts such as public services or industrial training, where familiarity with immersive systems may vary widely. In general, the system demonstrated a positive balance between accessibility, control, and comfort; however, opportunities remain for optimization, particularly in reducing momentary frustrations and managing perceived time pressure more effectively.

**Figure 15:**
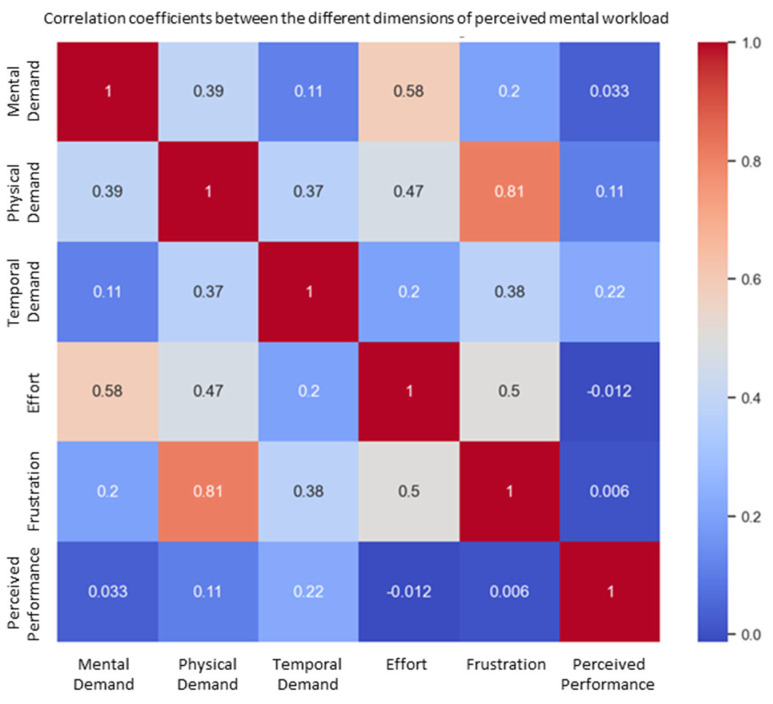
Correlation matrix between the different dimensions of perceived mental workload. The color intensity indicates the strength of the correlation.

A correlation analysis between the NASA-TLX dimensions revealed a notable positive relationship between frustration and physical demand, as shown in Figure 15. This suggests that users who experienced higher physical strain were also more likely to feel frustrated.

**Figure 16:**
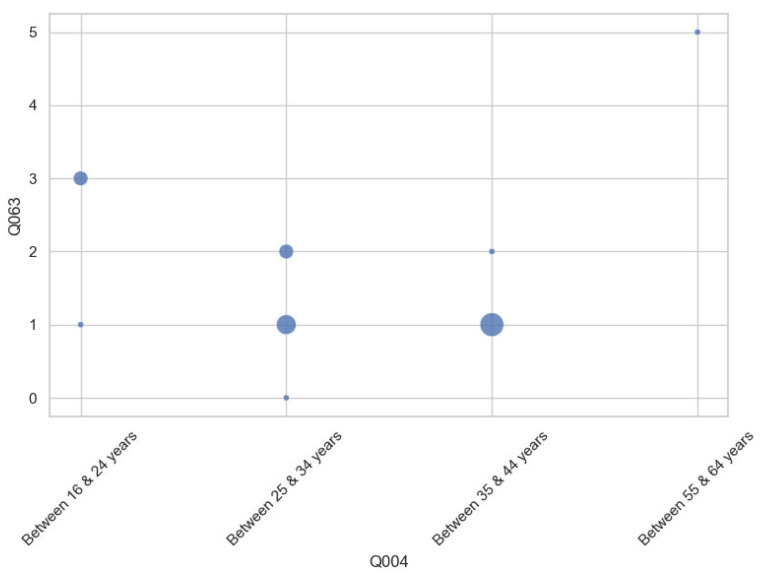
Distribution of perceived physical demand scores on a 0–10 scale (Q063) segmented by age group (Q004).

**Figure 17:**
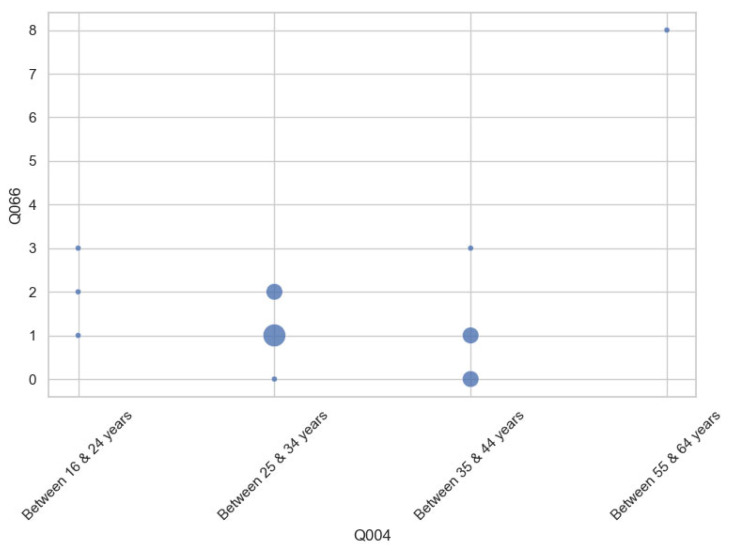
Distribution of perceived frustration scores on a 0–10 scale (Q066) segmented by age group (Q004).

Furthermore, inferential statistical tests (Student’s t-test and one-way ANOVA) were conducted to examine the significant differences across user subgroups. Two variables showed statistically significant differences by age group: physical demand and frustration. Figure 16 shows that older participants reported higher levels of physical demand, and Figure 17 shows the same relationship with frustration. Larger dot sizes represent a greater number of responses within those categories. Results indicate that age may influence the effortfulness or demand of a task.

**Figure 18:**
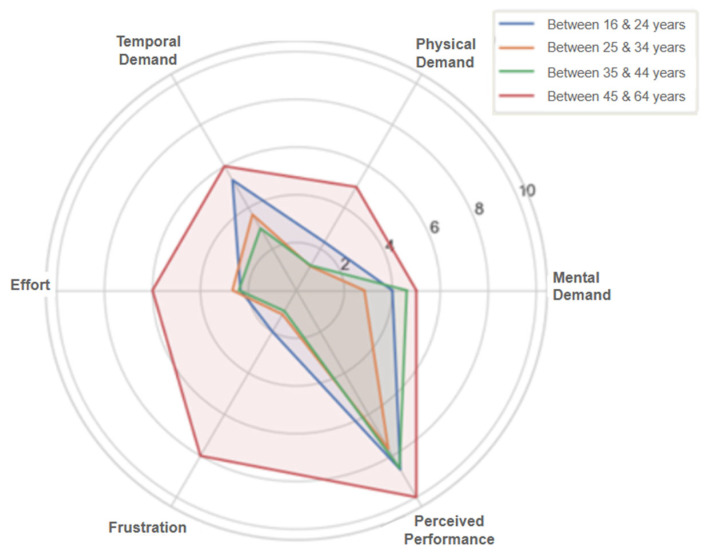
Mean NASA-TLX mental workload scores segmented by age group.

**Figure 19:**
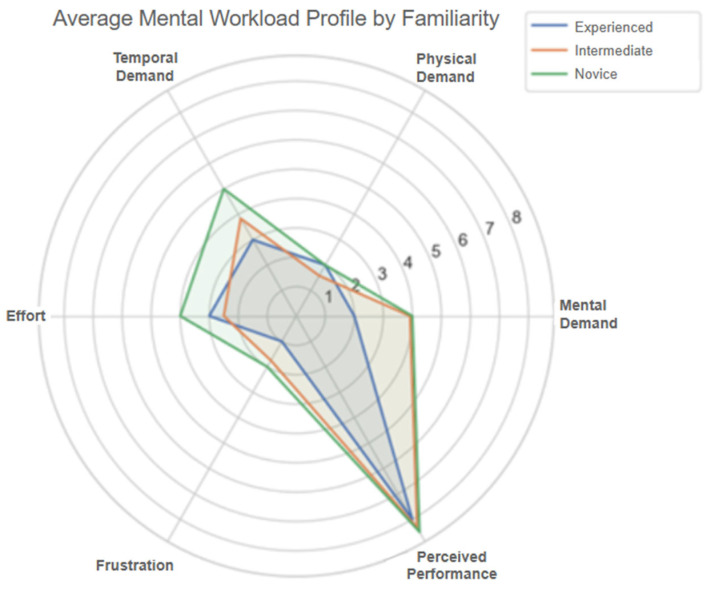
Mean NASA-TLX mental workload scores segmented by previous experience level.

Additionally, breakdowns of mental workload dimensions by age group (Figure 18) and prior experience level (Figure 19) reveal that older users and those classified as novice users perceived higher cognitive demand compared to intermediate or experienced participants. These insights further support the need for adaptable and supportive interface features, such as onboarding aids and customizable control modes, to ensure a consistent and inclusive experience for all users.

### 6.7. Trust and Overall Evaluation

The results indicate a high degree of trust in teleoperation systems among participants. In response to the question “Did you feel safe while remotely operating the robot?” The average score was 4.73 out of 5, with 13 out of 15 participants selecting the highest rating. This suggests that the system was perceived as being reliable, stable, and understandable. A single outlier (score of 2) highlights the need for further refinement in aspects such as gestural control accuracy or communication of system states; however, the overall trend points to high user confidence in the remote operation environment.

The overall experience with the system was also positive, with a mean rating of 4.8 out of 5 and a median of 5. Most users (13 out of 15) rated their experience with the highest possible score. Only two participants gave lower ratings (4 and 3), suggesting isolated areas for improvement but no widespread dissatisfaction. These results reflect a high level of user satisfaction with the interface design, system functionality, and the task itself.

**Figure 20:**
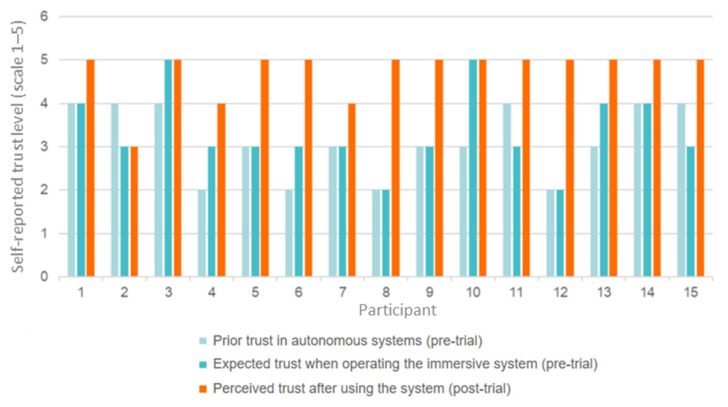
Self-reported trust levels in autonomous systems gathered at different moments of the experiments (1–5 scale) for each participant (*x*-axis shows the participant number).

Notably, the participants’ initial expectations of trust were more cautious, as shown in Figure 20. Pre-tests on trust in autonomous systems scored an average of 3.13 out of 5. Moreover, the expected trust in the immersive interface scored 3.33 out of 5 in the same tests. After the session, trust ratings increased significantly to 4.73, indicating that hands-on interaction with the system substantially improved participants’ confidence. All standard deviations were below one point, reinforcing the consistency of these positive assessments.

This increase in trust was also reflected in the participants’ answers about the broader perception of autonomous systems, as 73% reported that their perception of such systems had significantly improved following the experience, 13% indicated a slight improvement, while the remaining 13% saw no change.

### 6.8. Objective Evaluation Based on System Metrics

System-generated metrics were analyzed across tasks T02, T03, and T04, excluding T01 (familiarization stage). A total of 45 task executions were evaluated (15 participants performing three tasks each), considering successful completion without collision as the primary performance indicator.

**Figure 21:**
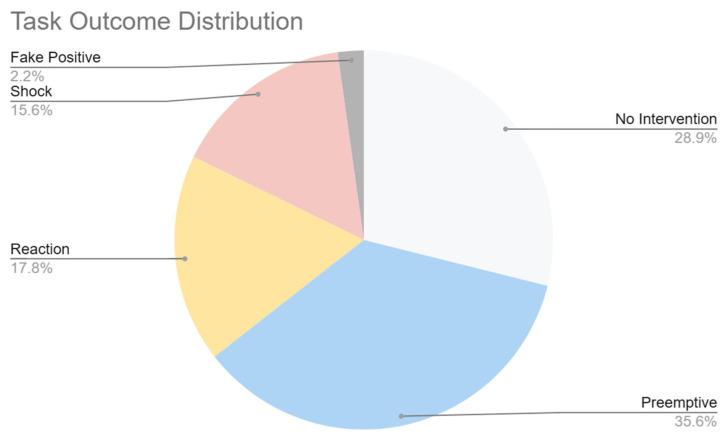
Distribution of task execution outcomes across the 45 evaluated trials (T02–T04).

Figure 21 shows the distribution of the task execution outcomes. “Shock” represents an intervention failure, while the remaining categories are considered favorable. Overall, the participants successfully completed 84.4% of the tasks, indicating a strong understanding of the environment and an effective capacity to respond to critical events. This high success rate suggests that the immersive teleoperation system provides an intuitive interface and supportive context for remote control under stress.

#### 6.8.1. Anticipatory Behavior and Intervention Patterns

In T02, which was designed not to require intervention, a single false positive was recorded, likely stemming from excessive user caution. In T03 and T04, an additional 16 false positives were detected. These were classified as preemptive interventions, where the participants acted before an emergency was triggered, successfully avoiding potential collisions. This anticipatory behavior was particularly notable among five participants, highlighting individual differences in control strategies that could inform the development of adaptive systems tailored to specific user profiles.

#### 6.8.2. Interface Type and Task Complexity

A progressive increase in collisions was observed in line with task complexity and control modality:

- T02 (no user intervention required): One participant experienced a collision.
- T03 (manual intervention using physical buttons): Two participants failed to prevent collisions.
- T04 (gesture-based control in an immersive environment): Four participants collided with obstacles.

These results suggest that while gesture-based interaction enhances immersion and realism, it also introduces greater challenges in terms of precision, gesture recognition, and response time, which can compromise effectiveness in high-stakes conditions.

#### 6.8.3. Global Collision Distribution

Globally, 60% of the participants completed all tasks without collision, indicating a high level of usability and adaptability. The remaining 40% experienced at least one collision, although most incidents were isolated. Only one participant exhibited multiple failures, with one collision in T03 and two in T04. This finding reinforces the need to improve gesture recognition systems and account for motor coordination and user variability in the design of critical teleoperation systems.

#### 6.8.4. Preliminary Response Time Analysis

The analysis of reaction times was limited to reactive interventions (i.e., those occurring after an emergency was triggered). However, due to a system logging issue—where timestamps were recorded with second-level rather than millisecond-level precision—a detailed analysis was not feasible. Nevertheless, a rough estimation was possible:

- In T03 (physical button-based control), two reactive interventions were recorded, with response times of <1 s and 1–2 s.
- In T04 (gesture-based control), six reactive interventions were detected: two occurred in less than 1 s and four within 1–2 s.

These preliminary results indicate that both control modalities enable timely reactions, although a more granular logging mechanism is required in future experiments to enable finer comparisons between the interaction types and user responsiveness.

### 6.9. Limitations

This study has several limitations that should be considered when interpreting the results.

Technical Constraints. A major technical limitation was encountered in the NVIDIA Isaac Sim environment. Despite its advanced capabilities for robotic simulation, Isaac Sim does not support the transmission of compressed image messages via ROS, which is a critical feature for real-time video streaming from onboard cameras. Initial attempts using uncompressed “Image” messages resulted in latency levels exceeding 300 ms, which is above the acceptable threshold for real-time systems, as established in the surgical robotics literature. Furthermore, the frame rate was inconsistent and introduced a jitter. To mitigate this, we implemented an alternative solution using FFMPEG and an RTSP server to stream the entire simulation viewport from a secondary monitor. Although this workaround enabled near-real-time feedback, it significantly diverged from standard ROS-based teleoperation pipelines and may have introduced limitations in scalability and system modularity. Notably, this limitation does not affect physical robot instances. In hindsight, if this constraint had been known earlier, alternative simulation platforms such as Gazebo or Unity may have been selected.

Gesture Recognition and Wearables. The gesture recognition system used in the immersive interface exhibited reduced accuracy when the participants wore haptic gloves, particularly under suboptimal lighting conditions. Although these gloves provide vibrotactile feedback and enhance embodiment, the trade-off in command recognition fidelity raises questions regarding their overall benefit in time-critical tasks. In the current experiment, haptic gloves were not used because the setup prioritized the acquisition of biometric variables (e.g., heart rate and electrodermal activity) for a parallel user study. This decision removed an important feedback channel from the interaction loop and may have influenced the user’s behavior and perceived system responsiveness.

Participant Profile. The study involved a total of 15 participants, which is a commonly accepted sample size for preliminary usability evaluations. However, the sample was not representative of the general population, as it consisted mainly of researchers and technology-savvy individuals from a single institutional group, many of whom had prior exposure to immersive systems or robotics. This potentially introduces bias in both subjective and objective performance measures, potentially overestimating usability, learnability, and trust compared with naive users or individuals with different demographic or cognitive profiles.

Simulation Environment. All tasks were conducted in a virtual simulation, which, although visually and functionally realistic, does not fully replicate the physical constraints, uncertainty, and environmental variability of real-world robotics. Therefore, generalization to physical deployment scenarios should be performed with caution, especially in terms of latency, input precision, and sensor reliability.

Data Resolution Limitations. The final limitation relates to the granularity of the system logs: timestamps were recorded with second-level precision, preventing a detailed analysis of reaction times in milliseconds, which is crucial for evaluating user responses in emergency scenarios. Future implementations should address this through high-resolution logging for a finer-grained analysis of user behavior.

Despite these limitations, the findings provide valuable insights into the feasibility and user perception of immersive teleoperation systems and offer a solid foundation for future improvements in both system design and experimental protocols.

## 7. Conclusions and Future Work

This study presents the design and evaluation of an immersive teleoperation interface for autonomous vehicles, which combines real-time simulation, virtual reality, and multimodal feedback to support human-machine collaboration in complex environments. The system was developed using a modular architecture based on ROS 2, enabling seamless transition between simulation (via NVIDIA Isaac Sim) and real-world deployment. This continuity not only accelerates development but also enhances reproducibility and reduces the risks typically associated with field testing.

Our findings suggest that immersive teleoperation can provide users with a perceived sense of control, satisfaction, and safety. Multimodal feedback (such as visual, auditory, and haptic channels) has proven instrumental in supporting situational awareness without adding excessive cognitive load. Furthermore, the ability to interact directly in an immersive environment significantly contributes to enhancing trust in autonomous systems. In this regard, the interface supports a human-in-the-loop paradigm, allowing human operators to intervene in edge cases in which fully autonomous systems may struggle. This human layer acts as both a safety mechanism and an ethical safeguard in the deployment of self-driving technologies.

Despite its strengths, the system also highlights the known challenges of immersive teleoperation. Latency, gesture recognition reliability, and learning curves for novel interaction paradigms remain technical and design aspects that warrant improvement. Notably, although gesture-based control was valued for its realism and naturalness, it was more error-prone and less precise than physical controls. These findings reinforce the importance of flexible interaction models that allow users to select control modes that match their preferences and capabilities.

Several design principles for future human-centered teleoperation systems emerged from this work:

- Multimodal feedback can effectively support decision-making without increasing the cognitive load.
- Interaction paradigms should be designed to accommodate different user preferences and capabilities.
- Gesture controls can be perceived as more intuitive and direct and may benefit from semantically meaningful and/or customizable actions with support mechanisms, such as visual or auditory cues, to aid memory and reduce fatigue.
- Immersive simulation is a powerful tool for the safe design and testing of interaction strategies in repeatable scenarios before deployment in real vehicles.

Future work will focus on deploying the system with physical autonomous platforms in order to evaluate its performance under real-world conditions. The ROS 2-based communication layer ensures that the control commands and telemetry data are processed in a standardized format, regardless of their origin. As a result, interfacing with a real robotic platform (provided it features a sensor suite similar to the one used in the simulation) will require only minimal ROS 2 topic remapping. Additionally, the configuration of feedback mechanisms (e.g., vibration thresholds, audio cues, and visual alerts) must be adapted to match the physical characteristics and operational context of the real robot, ensuring that the multisensory support remains effective and non-intrusive. In parallel, improvements to the gesture-based interface will be explored, including the design of more natural and semantically intuitive gestures, and the integration of user-defined control mappings. The predictive capabilities of the system for collision risk will also be refined by incorporating a real-time analysis of motion trajectories that take into account the speed, turning radius, and physical dimensions of the robot to generate alerts only when an obstacle intersects the projected driving path. Moreover, future experiments will aim to expand the participant pool to include more diverse user profiles, allowing the system to be validated and tailored to a broader range of cognitive and physical characteristics.

These preliminary results support the idea that immersive systems can significantly enhance human-robot interaction (HRI) in teleoperation scenarios. Ultimately, this research contributes to the development of safer, more trustworthy, and human-centered autonomous mobility solutions. Ensuring that remote operators can act confidently and effectively in high-risk contexts is essential for increasing public acceptance and enabling societal integration of autonomous technologies.

## Figures and Tables

**Table 1:** Messages and data via ROS2 topics.

Variable	Data Source	Message Type
Front Image	RGB Camera	sensor_msgs/msg/CompressedImage
360° Proximity	2D LIDAR	sensor_msgs/msg/LaserScan
1D Proximity	ToF laser	sensor_msgs/msg/Range
Temperature	Temperature sensor	sensor_msgs/msg/Temperature
Position	GPS	sensor_msgs/NavSatFix
Vehicle tilt	IMU	nav_msgs/Odometry
Linear and angular velocity	Odometry	nav_msgs/Odometry
Battery charge	Battery	sensor_msgs/msg/BatteryState
Camera pan & tilt	Joystick	sensor_msgs/msg/Joy
Movement	Joystick/Gestures	geometry_msgs/Twist

## Data Availability

Data is unavailable due to privacy restrictions.
